# Race, Gender, and the Development of Cross-Race Egalitarianism

**DOI:** 10.3389/fpsyg.2020.01525

**Published:** 2020-07-10

**Authors:** Sarah E. Gaither, Joshua D. Perlin, Stacey N. Doan

**Affiliations:** ^1^Department of Psychology and Neuroscience, Duke University, Durham, NC, United States; ^2^Samuel DuBois Cook Center on Social Equity, Center on Health and Society, Duke University, Durham, NC, United States; ^3^Department of Psychological Science, Claremont McKenna College, Claremont, CA, United States

**Keywords:** racial constancy, gender, egalitarianism, intergroup relations, child development

## Abstract

Over the course of development, children acquire adult-like thinking about social categories such as race, which in turn informs their perceptions, attitudes, and behavior. However, children’s developing perceptions of race have been understudied particularly with respect to their potential influence on cross-race egalitarianism. Specifically, the acquisition of racial constancy, defined as the perception that race is a concrete and stable category, has been associated with increased awareness of racial stereotypes and group status differences. Yet, little work has investigated behavioral outcomes stemming from the acquisition of racial constancy beliefs. Here, we investigate whether the presence or absence of racial constancy beliefs differentially predicts inequality aversion with racial ingroup versus outgroup members for young children. White children (*N* = 202; ages 3–8) completed three sticker resource-allocation games with either a White or a Black partner shown in a photograph, after which racial constancy was measured. Results revealed that the acquisition of racial constancy interacted with partner race to predict inequality aversion outcomes in one game; however, age and gender also exerted strong effects.

## Introduction

Children exhibit a range of social behaviors and preferences as early as the first year of life such as sharing their toys (Svetlova et al., 2010; Schmidt and Sommerville, 2012) time, and resources to help others (Warneken and Tomasello, 2006). However, children’s motivation to resolve inequality can be moderated by a number of factors, including children’s sharing options (Fehr et al., 2008) the gender of the child or the sharing recipient (Zimmer-Gembeck et al., 2005; Dunham et al., 2011) and a child’s temperament and parenting style (Russell et al., 2003).

Of particular importance in inequality aversion outcomes is the child’s relationship with the partner in question (Birch and Billman, 1986; Maccoby, 1990; Rose and Asher, 2004; Chernyak and Kushnir, 2013; Li et al., 2017). For example, children are less likely to be egalitarian with outgroup members (Fehr et al., 2008; Dunham et al., 2011; Weller and Lagattuta, 2013). However, testing egalitarian beliefs with racial outgroup members has been relatively understudied. Specifically, we do not know developmentally when or how awareness of race as a social category may shift children’s cross-race egalitarian behaviors. Past work shows that as children develop, they learn to adopt adult norms regarding the social construction of race. This change in social perceptions directly affects children’s endorsement of racial biases and stereotypes (Aboud, 1988; Bigler et al., 2001; Cameron et al., 2001; Nesdale and Flesser, 2001; Baron and Banaji, 2006) which in turn, may influence their egalitarian behavior toward racial ingroup versus outgroup members – the empirical question we test here. We expect that as children learn more about the social construction of race – and, in turn, consider race as a fixed social concept – children will show increased racial ingroup preferences and decreased racial outgroup inequality aversion.

Understanding children’s cross-race inequality aversion may lend critical insights into the emergence of either positive or fraught adult race relations. Thus, in the current study we examined children’s allocation of resources for both racial ingroup and racial outgroup members. We extend previous work by specifically investigating the social-developmental underpinnings of cross-race egalitarian concerns. In this paper, we focus on the acquisition of racial constancy (the perception of race as an immutable characteristic) as a developmental predictor of shifting children’s cross-race egalitarian perceptions. Additionally, we test whether gender, which has previously been shown to differentially impact children’s egalitarian beliefs, interacts with racial constancy endorsements. In the sections that follow, we discuss our reasons for emphasizing these two social-developmental factors in predicting cross- or same-race egalitarian behaviors.

### Racial Constancy Development and Cross-Race Egalitarianism

Inequality aversion is likely to be influenced by children’s *essentialism* – the tendency to think of social categories as fixed, unchangeable, and informative (Medin and Ortony, 1989; Gelman, 2003). If, as a child, I think that my social group is better or more important than another, that should surely influence my egalitarian behaviors toward that lower status outgroup. Developmental work on essentialism shows that children use essentialist views to categorize others by both race and gender by age four (Hirschfeld, 1995; Rhodes and Gelman, 2009; Pauker et al., 2010; Gaither et al., 2014). Importantly, psychological essentialism is multi-faceted and measures multiple ways children see different types of categories, including gender and race (Medin and Ortony, 1989; Gelman, 2003, for reviews). Here we focus on *racial constancy*, which represents one specific component of essentialist beliefs – that *race* is an unchangeable category (Rothbart and Taylor, 1992).

Children’s development of essentialized social categories, particularly regarding race, are known to lead to the development of normative intergroup biases and perceptions (Semaj, 1980; Hirschfeld, 1995; Levy and Dweck, 1999; Rhodes and Gelman, 2009; Pauker et al., 2010; Gaither et al., 2014). This, in turn, predicts increased levels of stereotyping toward racial and ethnic minority outgroups, particularly by dominant group members (White individuals; Levy et al., 1998; Levy and Dweck, 1999; Haslam et al., 2002; Rutland et al., 2005; Williams and Eberhardt, 2008; Pauker et al., 2010). These findings align with seminal psychological research demonstrating that all children regardless of their racial/ethnic background tend to learn a “White is good” bias early in development (Clark and Clark, 1947; Spencer, 1988; Hirschfeld, 2008) which directly influences their treatment of racial/ethnic minority group members. Thus, once children believe that their own and others’ racial group memberships are fixed and permanent, they will then be much more likely to also seek out either perceived similarities within, or differences between, racial ingroups and outgroups (Cameron et al., 2001; Rutland et al., 2005).

Moreover, these attitudes also manifest behaviorally. For instance, Leman and Lam (2008) showed that African Caribbean, South Asian, and White children often prefer playmates with a majority group membership (i.e., White). However, in terms of egalitarian behaviors, White children are more egalitarian with other White children compared toBlack children, highlighting an ingroup behavioral bias (Zinser et al., 1976, 1981; Zimmerman and Levy, 2000; Weller and Lagattuta, 2013). Taken together, this body of work suggests that children’s developing racial essentialism beliefs in combination with their own racial group membership and status in society may influence their egalitarian behaviors for racial ingroup versus outgroup members.

However, despite ample research demonstrating a link between essentialist beliefs and prejudiced attitudes (Levy et al., 1998, 2006; Levy and Dweck, 1999; Haslam et al., 2002; Rutland et al., 2005; Williams and Eberhardt, 2008; Pauker et al., 2010) research measuring how the acquisition of racial constancy may predict social outcomes such as inequality aversion has been overlooked (Stürmer and Snyder, 2009; Abrams et al., 2015). Since racial constancy – a touchstone of adult-like thinking about race – is generally associated with increased levels of stereotyping (and has also been studied primarily with White, high status children to date), one might expect racial majority group children who gain these adult-like views about race to be less egalitarian toward racial minority outgroup children and to show a stronger preference for egalitarian choices with other White racial ingroup members.

In an increasingly racially and ethnically diverse nation U.S. Census (2012) it is now both theoretically and practically important to investigate the impact of developmentally adopting adult-like views about race on intergroup behavior during early childhood, particularly for White majority children. Knowing egalitarian preferences are important for fostering positive intergroup relations (Fong et al., 2006) we set out to understand how racial constancy acquisition – one of the early developmental features of racial essentialism – influences White children’s interactions with racial ingroup (White) versus outgroup (Black) members.

### Gender and Cross-Race Egalitarianism

In addition to race, gender is another social identity that is likely to independently, and in interaction with race, influence egalitarian behavior. Although gender bias is well established in children across a variety of dimensions (for reviews, see Ruble and Martin, 1998; Miller et al., 2006) there remains mixed evidence supporting gender differences and egalitarian behaviors. Specifically, some findings highlight an absence of a gender differences regarding egalitarianism (Renno and Shutts, 2015; Sierksma et al., 2018) some work shows girls have a stronger ingroup bias (Dunham et al., 2011) and other work, finds that girls tend to behave more egalitarian than boys overall (Maccoby, 1990; Chung and Asher, 1996; Eisenberg and Fabes, 1998; Rose and Asher, 2004; Rose and Rudolph, 2006). However, none of this past work assessed race in addition to gender within egalitarian decision contexts.

However, one study examining children’s perceptions of unequal allocations of stickers found that more girls than boys made choices to rectify perceived unequal sticker allocations (LoBue et al., 2011) suggesting girls may be more aware or cognizant of status group differences within egalitarian-based contexts. Moreover, past work suggests that White girls are more willing than White boys to engage prosocially with Black children (Zimmerman and Levy, 2000). Additionally, boys generally possess stronger explicit racial biases and gender ingroup biases than girls (Signorella et al., 1993; Baron and Banaji, 2006) with White girls tending to be more likely than White boys in attempting to ameliorate the learned status difference that Black individuals occupy a lower status in society (Nesdale and Flesser, 2001; Bigler et al., 2003; Pauker et al., 2010; LoBue et al., 2011). Knowing these social perceptions remain consistent throughout adulthood (Johnson and Marini, 1998; Eagly et al., 2004; Hausmann and Ryan, 2004) research needs to better understand the developmental origins of these biased behaviors (Sutter, 2007; Fehr et al., 2008).

### The Current Study

The present study had two primary questions: (1) does the developmental acquisition of racial constancy beliefs influence cross-race sharing behavior in young White children? and (2) does gender influence cross-race egalitarian behaviors for White children? We used a previously established inequality aversion paradigm (see Fehr et al., 2008 for full details on method development), which involved resource allocation using stickers in three games to measure the concern for the welfare of others across racial group lines (see the “Materials and Methods” section for a more detailed description). We tested this paradigm with White children ages 3–8 years – the age-range in which White children typically begin to exhibit racial constancy beliefs and endorse stereotypes (Bigler et al., 1997; Quintana, 1998; Rhodes and Gelman, 2009; Pauker et al., 2010; Gaither et al., 2014) as well as the age range assessed by Fehr et al. (2008). Moreover, White children have been studied most often in cross-race perception work because of their majority high status position in society. Children were randomly assigned either a White or Black, male or female partner for all three games, after which their racial constancy beliefs were measured. Importantly, we included Sharing Partner Gender in the model to account for its potential effects, but our real interests were focused of Sharing Partner Race and Racial Constancy effects.

Based on theories of ingroup favoritism (Tajfel, 1974) we predicted that White children’s emerging racial constancy beliefs would result in diminished egalitarian tendencies with racial outgroup members. Specifically, we hypothesized that children would be less egalitarian with cross-race sharing partners after the adoption of racial constancy compared to before developmentally adopting these beliefs. We did not have any *a priori* hypotheses regarding how results from each game would differ. The three games selected individually measured distinct egalitarian behaviors, serving as a robust test of the role racial constancy knowledge may play in shaping cross-race egalitarian choices. Additionally, based on previous research, we expected that girls would be more egalitarian than boys regardless of the group membership of their partner, and that boys may be less egalitarian with racial outgroup members in comparison to girls.

## Materials and Methods

### Participants

Since our methods were directly adapted from Fehr et al. (2008) their sample size of 40 children per condition was also used as a recruitment guide for the present study. Since we were interested in the effects of both participant gender and sharing partner race on egalitarian behaviors, our recruitment goal was 200 White children, which would give us approximately 50 children per cell and variation regarding racial constancy endorsements. Notably, this study surpassed the sample size from previous egalitarian-focused child experiments (Fehr et al., 2008; Dunham et al., 2011; Renno and Shutts, 2015). Additionally, since we were interested in testing how racial constancy endorsement may shift egalitarian behaviors for racial ingroup versus outgroup partners, White children (*N* = 202; 59.4% female; age range: 3–8 years, *M*_*age*_ = 4.97, *SD* = 1.30) were recruited from two schools (*n* = 24) and a museum science center (*n* = 178) in the greater Boston area from 2012–2013. Parents were informed about the study, including its focus on race, from either a letter sent home by the school administration (25% response rate) or through an in-person invitation to participate at the science center (85% response rate). Parents at the science center were asked not to interfere with the testing session and to watch from behind the child so that social referencing would not affect our results. Parents at the science center also confirmed the child’s demographic profile through a short survey administered on site. Based on parent-reported demographics from the school’s returned consent form, as well as the science center’s data on the average visitor, approximately 68% of our participants were from families earning $75,000 or more per year and approximately 75% were from families in which at least one parent had a college degree.

### Measures and Procedure

For participants recruited from schools, parents completed an optional demographic form. At the science center, parents were asked this in-person. After receiving parental consent, the experimenter asked for children’s verbal assent and made clear that the child could stop at any point. Children completed the study in either an area separate from the classroom or an area separate from other children at the science center. Each participant completed two tasks: a sticker allocation task and a racial constancy task. To avoid carry-over effects from the racial constancy measurement which explicitly asked about race, the sticker task always came first and then the racial constancy measurement. The three sticker games were counterbalanced across participants to ensure no order effects.

#### Sticker Task

**FIGURE 1:**
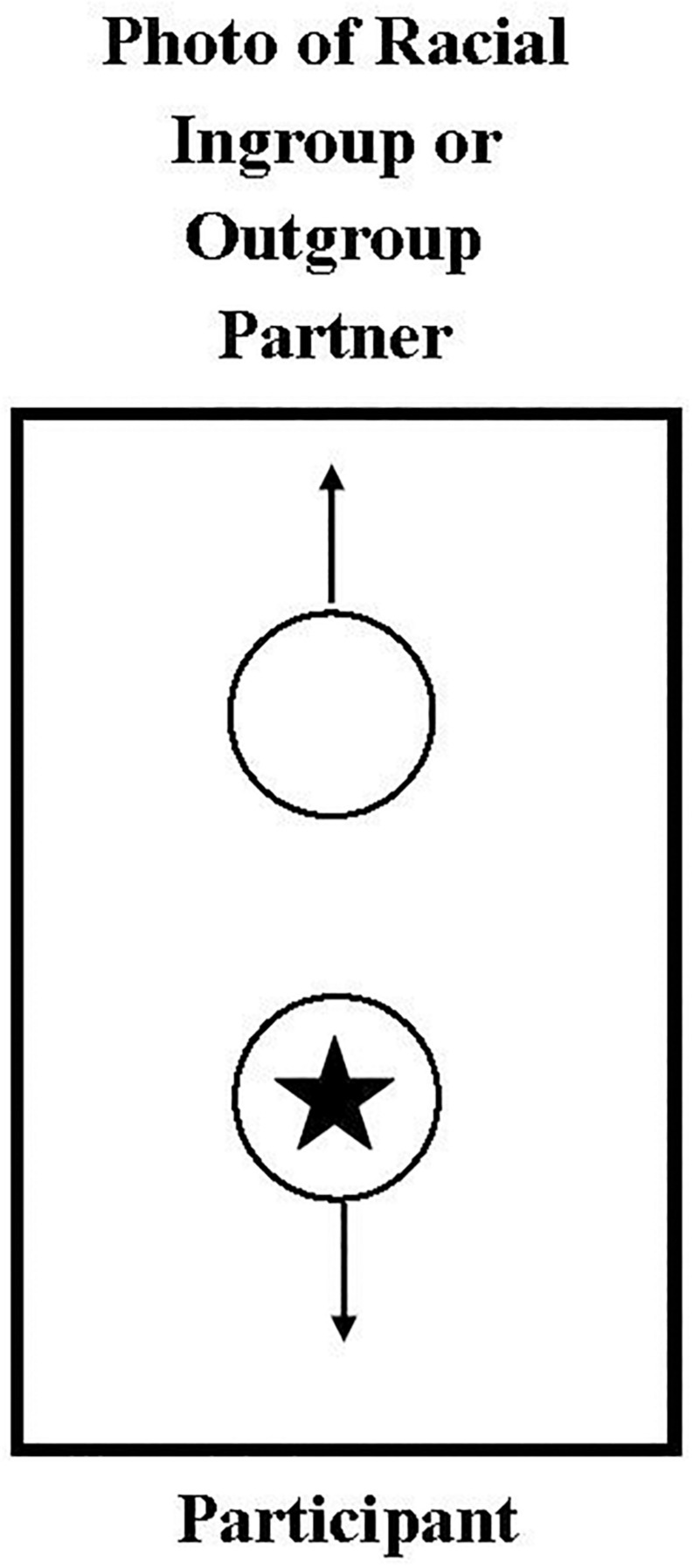
Image of cardboard setup illustrating a (1,0) choice where the participant gains one sticker and their partner gains zero.

Using methods directly adapted from Fehr et al. (2008) participants were seated in front of two cardboards with two circles and arrows on them (see Figure 1). One arrow pointed to the participant, indicating that the stickers in that circle would go to them, while the other arrow pointed to a photo of either a White or Black child. To ensure generalizability, one of twelve color photos of a White (*n* = 6, racial ingroup) or Black (*n* = 6, racial outgroup) smiling girl or boy was used across all participants. These photos were pretested by adults (*N* = 20) to be equivalent on perceived age (mean perceived age was around 5 years to equate with the middle age of our participant sample), affect, attractiveness, and perceived racial group membership. Children had the same partner across both the training and target trials. Therefore, children were randomly assigned to a 2 (partner race: White, Black) × 2 (partner gender: male, female) between-subjects design.

To create a more realistic interaction, participants were given an envelope and told: “You will put any stickers from this circle [*pointed to it*] that is pointing toward you in your envelope. And I will put any stickers from this circle [*pointed to it*] that is pointing to the other child in their envelope for us to give them when they come back to our lab” (see Figure 1). Participants then made three choices across three different games with three different types of stickers to ensure engagement with the task. In each game, participants chose between two options that were depicted on the cardboards (left board and right board choices were counterbalanced) and each game was explained in detail to the participant to ensure the participant completely understood the associated outcomes. Following Fehr et al. (2008) Game 1 (prosocial trial) asked participants to choose between an allocation of (1,1) – one sticker for themselves and one for their partner – and an allocation of (1,0) – one sticker for themselves and zero for their partner. Thus, participants can give a sticker to their partner at no cost to themselves. Game 2 (envy trial) presented (1,1) and (1,2) allocation choices. Although for both choices the participant gains one sticker, the (1,2) option gives their partner an extra sticker (i.e., disadvantageous inequality), and thus can promote envy. Game 3 (sharing trial) presents (1,1) and (2,0) allocations, where choosing to share equally with one’s partner (1,1) comes at a cost to one’s self (i.e., advantageous inequality). In other words, selfish children should never choose the (1,1) allocation (see Fehr et al., 2008 for more detailed game definitions). After the three games, the experimenter asked participants which sticker they liked the best and the least to ensure sticker types did not influence allocation selections.

#### Racial Constancy

After the sharing task, participants completed a three-item measure adapted from previous tasks to examine children’s racial constancy beliefs (Semaj, 1980; Hirschfeld, 1995; Tyler, 2000; Ruble et al., 2007; Pauker et al., 2010; Gaither et al., 2014). In the first item, children were shown three faces (all photos were matched to the child’s gender). A photograph of a Black or White child was placed above a photograph of a Black adult and a photograph of a White adult. The experimenter asked, “When this child grows up, will they look more like this adult [White] or that adult [Black]?” (the order of the adult photos was counterbalanced across all participants). In the second item, children were shown a similar picture array except they now saw either a Black or White adult pictured above a Black child and a White child (in counterbalanced order). The experimenter then asked, “When this adult was little, did they look more like this child [White] or that child [Black]?” Finally, the experimenter pointed to the picture of a White child and asked, “If this child really wanted to be Black and change his/her skin color could he/she do that?” Children then explained *why* they believed that the child could or could not change their racial group membership. Two coders rated all children’s responses to this question in order to examine their reasoning and understanding of racial group membership with 100% agreement.

Children were categorized as having racial essentialist thinking if they: (a) correctly made a race match in the first two questions and answered “no” to the last question, indicating that they believe race is both stable across the lifespan and immutable, and (b) utilized essentialist reasoning in their explanation for why someone could not change their skin color (referencing either immutability, i.e., “you can’t change your skin color”; inheritability/biology, i.e., “she looked that way when she was born”; examples of responses clearly not showing racial constancy endorsement included phrases such as “maybe painting her face” or “she could get a crayon.” These responses were coded as incorrect reasoning since they show the child does not consider race a stable trait; see Ruble et al., 2007; Pauker et al., 2010; Gaither et al., 2014 for similar coding strategies). If children did not provide reasoning for their answer or if that reasoning did not fall into any of the above categories (e.g., “they like it like that”), they were coded as not having racial constancy. In other words, children needed to answer all questions correctly and provide essentialist reasoning to be coded as having racial constancy understanding. Therefore, children were divided into two groups: non-essentialist and essentialist. Investigating children’s social reasoning by asking for their explicit reasoning gives us unique insight into children’s understandings of concepts not available through simple forced-choice measures (Killen and Stangor, 2001; Gimenez and Harris, 2002; Taylor et al., 2009) which is why children had to answer all questions correctly, in addition to providing supportive reasoning, to be designated as having adopted racial constancy beliefs. Importantly, each yes/no question leading up to the reasoning question does not increase in difficulty, which is why this particular measurement of racial constancy is measured categorically rather than continuously.

## Results

### Analytical Plan

A logit regression analysis was used to test our hypotheses, with the choices made in each of the three tasks as respective outcome variables in three different regression models. Our analyses focused on five primary predictor variables (Age, Participant Gender, Racial Constancy, Sharing Partner Race, and Sharing Partner Gender), and four two-way interaction terms (Racial Constancy by Sharing Partner Race, Participant Gender by Sharing Partner Race, Participant Gender by Sharing Partner Gender, and Age by Sharing Partner Race; see Table 1; all data is publicly available at https://osf.io/pngfm/. All analyses were conducted using IBM (2020) SPSS version 26. When interactions are included in logit models, *p*-values for main effects differ between parameter estimates and tests of model effects. This is because the latter examines overall effects, whereas parameter estimates examines the effects of one variable when the moderating variable is set to the reference category (UCLA IDRE Statistical Consulting Group, 2020). Given that we are interested here in examining overall effects across all levels of the moderating variable, we used the test of model effects to determine whether to report main effects as significant. However, we report both statistics in text for the purpose of transparency. Interactions show the same effects across both tests, so only parameter estimates are reported.

**TABLE 1:** Logit regression test of model effects predicting the likelihood of egalitarianism in Game 1 [(1,1) vs. (1,0)].

	Type III		
	
	Wald chi-square	df	*p*
Age	5.055	1	0.025*
Sharing partner race	1.121	1	0.290
Racial constancy	0.397	1	0.529
Participant gender	5.593	1	0.018*
Sharing partner gender	0.578	1	0.447
Sharing partner race*racial constancy	5.298	1	0.021*
Sharing partner race*age	0.023	1	0.878
Participant gender*sharing partner gender	2.375	1	0.123
Sharing partner race*participant gender	0.094	1	0.759

*p < 0.050. For Game 1, (1,1) received the higher value. For Gender, Males received the higher value. For Racial Constancy, those with racial constancy received the higher value. For Sharing Partner Race, Black sharing partners received the higher value. For Sharing Partner Gender, Males received the higher value.

All nine of these terms were theoretically motivated. With respect to age, research indicates that altruism increases with age (Benenson et al., 2007) which is why we chose to use age as a continuous variable rather than a grouping variable like past work. Specifically, we wanted to assess if other developmental variables of interest explained variance that was unaccounted for by age. This was important in order to show that racial constancy – which emerges over the course of early child development – uniquely predicts cross-race egalitarian behaviors beyond the effects of age. The Racial Constancy by Sharing Partner Race interaction term was added to examine this moderation, and we were interested to test whether this moderation predicted egalitarian choices beyond any potential main effects of Sharing Partner Race. Further, an Age by Sharing Partner Race interaction term was included for two reasons: (1) to attempt to replicate the results of Fehr et al. (2008) and (2) to assess if the Racial Constancy by Sharing Partner Race interaction term remained significant even when including the Age by Sharing Partner Race term. Importantly, age was mean centered to avoid collinearity issues when interacted with Sharing Partner Race.

We included Participant Gender in the model because, as explained previously, we were interested in assessing if a Participant Gender by Sharing Partner Race interaction significantly predicted egalitarianism. The Participant Gender by Sharing Partner Gender interaction term was used as a potential confound to assess the likelihood that children would be more likely to be inequality averse with a same- than a cross-gender partner. However, gender ingroup sharing preferences were not found, which adds to the mixed findings regarding gendered prosocial outcomes; Dunham et al., 2011; Renno and Shutts, 2015).

Preliminary analyses showed that 43.6% of children had adopted racial constancy beliefs. Importantly, comparing boys and girls, there were no gender differences on the number of children who endorsed racial constancy beliefs, *t*(200) = 0.08, *p* = 0.875. Further, the average age of those with racial constancy (*M*_*age*_ = 5.08, *SD* = 1.16) was not significantly different from the average age of those without (*M*_*age*_ = 4.89, *SD* = 1.41); *t*(199.164) = −1.073, *p* = 0.285, Cohen’s *d* = 0.148), indicating no collinearity between these variables and that age is not simply a reason that children may understand more about race. As an additional test to assess collinearity, a point-biserial correlation between age and racial constancy was run and showed no association (*r* = 0.074, *p* = 0.297). Additionally, there was a linear relation between the continuous predictor variable age and the logit of Games 1, 2, and 3, as indicated by the Box-Tidwell procedure run separately for each outcome variable. Thus, our data met the assumption of linearity for all logit regression equations. We included all cases in the analyses regardless of standardized residual values, given that there was no other justifiable reason to exclude these cases based on experimenter notes. As further justification for including these cases, there were no formal exclusion criteria outlined prior to conducting the analyses. Finally, in preliminary explorations, excluding these outliers only increased the significance of both models and the significance of individual predictors; in the spirit of conservative analyses, we decided to report the results with the complete sample. Finally, there was no association between sharing behaviors and testing location (museum or lab) (Game 1: φc = 0.038, *p* = 0.866; Game 2: φc = 0.119; *p* = 0.244; Game 3: φc = 0.120, *p* = 0.234), so analyses were collapsed across location. In the sections that follow, we discuss the results for each game separately.

### Game 1 [(1,1) vs. (1,0)] – Prosocial Trial

Results for the logit regression are presented in Table 2. Our model was statistically significant [LR χ^2^(9) = 22.657, *p* = 0.007], meaning that our model better predicts variance in the outcome than a null model, that is, one without any predictors.

**TABLE 2:** Logit regression parameter estimates predicting the likelihood of egalitarianism in Game 1 [(1,1) vs. (1,0)].

	*B*	S.E.	Wald Chi-Square	*df*	*p*	Odds ratio	95% CI for Odds ratio	
							
							LL	UL
Age	0.326	0.229	2.024	1	0.155	1.385	0.884	2.170
Sharing partner race	1.416	0.689	4.223	1	0.040*	4.122	1.068	15.916
Racial constancy	0.657	0.518	1.607	1	0.205	1.928	0.699	5.320
Participant gender	0.400	0.654	0.373	1	0.541	1.491	0.414	5.371
Sharing partner gender	−0.852	0.529	2.590	1	0.108	0.427	0.151	1.204
Sharing partner race*racial constancy	−1.804	0.784	5.298	1	0.021*	0.165	0.035	0.765
Sharing partner race*age	0.048	0.311	0.023	1	0.878	1.049	0.570	1.930
Participant gender*sharing partner gender	1.143	0.742	2.375	1	0.123	3.137	0.733	13.425
Sharing partner Race*participant gender	−0.215	0.701	0.094	1	0.759	0.806	0.204	3.187

*p < 0.050. We do not report the significant effect of Sharing Partner Race in the text because we focus on significance value of the test of model effects. For Game 1, (1,1) received the higher value. For Gender, Males received the higher value. For Racial Constancy, those with racial constancy received the higher value. For Sharing Partner Race, Black sharing partners received the higher value. For Sharing Partner Gender, Males received the higher value.

**TABLE 3:** Cross-tabulations of racial constancy and sharing partner race with game 1.

	Game 1 prosocial task		
	
	(1,0)	(1,1)	Total
**Racial constancy**			
White partner	5 (13.2%)	33 (86.8%)	38
Black partner	15 (30.0%)	35 (70.0%)	50
**No racial constancy**			
White partner	18 (29.0%)	44 (71.0%)	62
Black partner	12 (23.1%)	40 (76.9%)	52
Total	50 (24.8%)	152 (75.2%)	202

Age was a significant predictor in the test of model effects [Type III Wald χ^2^(1) = 5.055, *p* = 0.025]; however, in the parameter estimate test, age was non-significant [Wald χ^2^(1) = 2.024, *p* = 0.155]. The general trend was for older children to be slightly more likely than younger children to make the egalitarian choice (*B* = 0.326, OR = 1.385). Similarly, Gender was also statistically significant in the test of model effects [Type III Wald χ^2^(1) = 5.593, *p* = 0.018], but was non-significant in the parameter estimate [Wald χ^2^(1) = 0.373, *p* = 0.541], with girls being slightly more likely than boys to make the egalitarian choice (*B* = 0.400, OR = 1.491). There was also a non-significant main effect of Sharing Partner Race in the test of model effects [Type III Wald χ^2^(1) = 1.121, *p* = 0.290], which was significant in the parameter estimate [Wald χ^2^(1) = 4.223, *p* = 0.040] suggesting Sharing Partner Race is not a strong enough predictor on its own for our outcome of interest. Finally, there was a significant Racial Constancy by Sharing Partner Race interaction [Wald χ^2^(1) = 5.298, *B* = −1.804, OR = 0.165, *p* = 0.021]. Pairwise comparisons revealed that children with racial constancy were more likely to be egalitarian with White ingroup partners than with Black outgroup partners [Wald χ^2^(1) = 5.629, *p* = 0.018]. Further, children with racial constancy shared more than those without when sharing with a White partner [Wald χ^2^(1) = 4.813, *p* = 0.028]. This interaction suggests a developmental shift in how children cognitively process and weigh decisions regarding egalitarianism with racial ingroup and outgroup partners.

### Game 2 [(1,1) vs. (1,2)] – Envy Trial

Our model was not statistically significant [LR χ^2^(9) = 12.733, *p* = 0.175, *N* = 201; note: there was one missing data point for this trial, resulting in a different *N*], indicating that our model did not predict variance in the outcome better than a null model, or a model without any predictors.

### Game 3 [(1,1) vs. (2,0)] – Sharing Trial

Results for the logit regression are presented in Tables 4, 5. The test of model effects for our model was statistically significant [LR χ^2^(9) = 24.236, *p* = 0.004], indicating that our model better predicted variance in the outcome than a null model, that is, one without any predictors. Age was the only statistically significant predictor [test of model effects: Type III Wald χ^2^(1) = 14.649, *p*<0.001; parameter estimate: Wald χ^2^(1) = 7.497, *B* = 0.592, OR = 1.808, *p* = 0.006], such that older children chose the (1,1) option – the egalitarian option – significantly more often than younger children. This finding is in line with past work demonstrating that older children are often more egalitarian than younger children.

**TABLE 4:** Logit regression test of model effects predicting the likelihood of egalitarianism in Game 3 [(1,1) vs. (2,0)].

	Type III		
	
	Wald chi-square	df	*p*
Age	14.649	1	0.000*
Sharing partner race	2.116	1	0.146
Racial constancy	0.016	1	0.898
Participant gender	1.791	1	0.181
Sharing partner gender	1.933	1	0.164
Sharing partner race*racial constancy	0.801	1	0.371
Sharing partner race*age	0.348	1	0.555
Participant gender*sharing partner gender	0.382	1	0.536
Sharing partner race*participant gender	0.677	1	0.410

*p < 0.050. For Game 3, (1,1) received the higher value. For Gender, Males received the higher value. For Racial Constancy, those with racial constancy received the higher value. For Sharing Partner Race, Black sharing partners received the higher value. For Sharing Partner Gender, Males received the higher value.

**TABLE 5:** Logit regression predicting the likelihood of egalitarianism in Game 3 [(1,1 vs. (2,0))].

	*B*	S.E.	Wald Chi-Square	*df*	*p*	Odds Ratio	95% CI for Odds Ratio	
							
							LL	UL
Age	0.592	0.216	7.497	1	0.006^∗^	1.808	1.183	2.762
Sharing partner race	1.023	0.623	2.694	1	0.101	2.780	0.820	9.428
Racial constancy	0.247	0.467	0.280	1	0.597	1.280	0.513	3.196
Participant gender	0.500	0.602	0.690	1	0.406	1.649	0.507	5.361
Sharing partner gender	−0.660	0.522	1.599	1	0.206	0.517	0.186	1.438
Sharing partner race*racial constancy	−0.576	0.643	0.801	1	0.371	0.562	0.159	1.984
Sharing partner race*age	−0.159	0.269	0.348	1	0.555	0.853	0.504	1.445
Participant gender*sharing partner gender	0.409	0.661	0.382	1	0.536	1.505	0.412	5.499
Sharing partner race*participant gender	−0.530	0.644	0.677	1	0.410	0.589	0.167	2.080

*p < 0.050. For Game 3, (1,1) received the higher value. For Gender, Males received the higher value. For Racial Constancy, those with racial constancy received the higher value. For Sharing Partner Race, Black sharing partners received the higher value. For Sharing Partner Gender, Males received the higher value.

## Discussion

To the best of our knowledge, this is one of the few studies to examine one potential developmental milestone that may influence children’s divergent egalitarian behaviors when considering a same-race vs. other-race partner. Specifically, we assessed the effect of racial constancy acquisition (a developmental milestone marking more adult-like thinking about race) and gender on cross-race egalitarian behaviors. Taken together, these findings provide some suggestive evidence that both the adoption of a racial constancy perspective and one’s gender influence egalitarian behaviors, particularly in cross-race egalitarian decision contexts between a White and a Black child. Although other demographic factors such as age also influence egalitarian behaviors, we provide some of the first developmental empirical evidence that highlights some of the behavioral downstream consequences that may stem from the adoption of racial constancy beliefs in White children. However, Game 2 (envy) did not show any differences based on our predictors. Compared to previous work which only involved preschool classrooms as an ingroup/outgroup marker (Fehr et al., 2008) perhaps envy contexts are processed differently with real-world groups such as race (Zimmerman and Levy, 2000; Weller and Lagattuta, 2013). Since Game 1 (prosocial) and Game 3 (sharing) were the only statistically significant models, we will focus primarily on the implications of these results in the sections that follow.

In the logit regression models for both Game 1 (prosocial) and Game 3 (sharing), age was a significant predictor. These results from two different games provide convergent evidence that children exhibit an increase in egalitarian tendencies as they mature. Indeed, younger children are, on average, more selfish than older children (Froming et al., 1983; Schmidt et al., 1988; Eisenberg and Fabes, 1998). This accords with past work finding that children value inequality aversion as they age (Fehr et al., 2008; Blake and McAuliffe, 2011). Interestingly, while Fehr et al. (2008) found a strong ingroup bias at ages 7–8 (only 12% of children in Game 3 made egalitarian choices with a preschool outgroup member), our results show that 77.8% of 7–8 year olds who were randomly assigned a racial outgroup sharing partner behaved in an egalitarian manner.

This suggests that perhaps past work has overlooked the potential social differences children may weigh when considering a minimal outgroup member. Specifically, designating a child as being from another preschool is bound to function differently than a societally constructed and socially meaningful outgroup like race (Fehr et al., 2008). Although this previous work did not measure how socially meaningful one’s preschool classroom identity may be, race is often a visible group marker that is extremely salient to young children (Aboud, 1988; Banaji et al., 2008; Dunham et al., 2008). Additionally, race, unlike one’s preschool classroom, is an unchangeable group membership which is an often-manipulated attribute in minimal group experiments (social mobility; Nesdale and Flesser, 2001; Nesdale et al., 2003). Moreover, research involving minimal ingroup paradigms with adults has also shown that lower levels of identification with an artificially created group can reduce commitments to that group (Jetten et al., 1996; Reynolds et al., 2000; Scheepers et al., 2006). Therefore, preschool classroom assignment may not hold the same social weight as a group like race or ethnicity. Therefore, our findings motivate the need for additional research comparing the effects of different types of group membership – groups with and without social identity relevance, groups that are more essentialized than others, and whether a child’s own self-identification with a given group influence egalitarian choices. Additionally, with other recent work suggesting that children’s ingroup bias within a minimal group paradigm also does not always predict egalitarian choices (Gonzalez et al., 2020) additional work testing these important boundary effects will allow us to more fully understand the role that various types of group membership plays in shaping social decisions across development.

In the regression model for Game 1 (prosocial), two additional predictor variables were significant: Participant Gender and the interaction between Racial Constancy and Partner Race. As expected, the acquisition of racial constancy predicted changes in cross-race sharing behavior. Overall, children who had adopted racial constancy were more likely to show egalitarian behaviors when interacting with a White partner than those who had not yet adopted racial constancy. Further, those with racial constancy were more likely to show egalitarian behaviors with White partners than Black partners. These results support our hypothesis that the acquisition of racial constancy would predict diminished egalitarian behaviors with racial outgroups, adding to past work stating that children’s use of race as a meaningful category can affect interracial social behavior beyond simply perceiving race (Aboud, 1988; Bigler et al., 2001; Pauker et al., 2010). Further, children showed greater ingroup favoritism after racial constancy acquisition (i.e., they were more egalitarian towards White children), which we were not necessarily expecting. Finally, these results remain even when controlling for both Age and an Age by Sharing Partner Race interaction, indicating the robustness of this finding. Thus, racial constancy influences cross-race egalitarianism beyond the effect of age more broadly, suggesting a specific developmental construct for cross-race egalitarianism differences. Importantly, given that actual in-person interracial interactions are difficult to run with child samples (confederates are the common method used in adult studies, which is not feasible for children), this paradigm with a realistic cross-race sharing encounter reflects one of the few to measure actual cross-race behavior in children.

Our results in Game 1 also lend some limited support to earlier work demonstrating that, on average, boys show fewer egalitarian behaviors than girls (Moore and Eisenberg, 1984; Lennon and Eisenberg, 1987; Maccoby, 1990; Eisenberg and Fabes, 1998; Zimmerman and Levy, 2000; Rose and Rudolph, 2006; Pursell et al., 2008). Based on gender-role socialization theory (Fabot and Hagan, 1991; McHale et al., 1999) this gender difference is thought to be due to children’s emerging endorsement of gender norms (e.g., girls as nurturing and boys as assertive in matters of self-interest), which in turn shapes their behavior. Girls are socialized to behave in more egalitarian ways and to prize considerateness of others’ needs, while boys are socialized to be independent and assertive of one’s needs, even if to the detriment of the needs of others (Fabot and Hagan, 1991; McHale et al., 1999). These socialization pressures may ultimately give rise to increased egalitarian attitudes, beliefs, and behaviors, which may begin early on in development as suggested by the present data. In other words, this developmental period might not only be indicative of the beginning of gender differences relating to egalitarian beliefs, but also may be the same period during which concern for one’s racial ingroup is developing. Future work should test a similar paradigm with younger children to pinpoint at what age gender (versus racial) constancy comes online and how that belief adoption differentially influences egalitarian behaviors across gender and racial group lines.

## Limitations and Future Directions

Although we focused here on the links between gender and egalitarian behaviors on the one hand and racial constancy and egalitarian behaviors on the other, additional social factors not measured in the present study may also contribute to children’s egalitarian choices. For example, children’s interracial exposure has been shown to predict their racial preferences and behaviors (Cross et al., 1971; De Heering et al., 2010). Although we do not have data about children’s cross-race contact here, Boston, MA has been consistently rated as one of the most racially segregated cities in the United States (Tucker, 2019) suggesting that White children in our sample likely do not have much contact with other Black individuals. Therefore, the lack of exposure to Black children (and potentially other racial outgroups as well) of the children in this sample may have affected our outcomes. Future work should recruit children with more racial diversity exposure to explicitly test this possibility. We would predict that children with more Black contact would show less of a racial ingroup egalitarian preference.

Additionally, in this study we only examined White children. Racial/ethnic minority children might show different sharing preferences due to their differential awareness of race and status and their need to navigate being a minority within a White majority nation (Aboud, 1988; Phinney, 1992; Tropp and Wright, 2003; Rockquemore and Laszloffy, 2005; Kinzler and Dautel, 2012). In fact, past work shows that racial/ethnic minority children often do not develop as strong of racial/ethnic ingroup preference due to balancing being a minority in a White majority society (Spencer, 1984; Ocampo et al., 1997; Dunham et al., 2007). On this point, we do not know whether racial minority members would exhibit these same patterns with racial outgroup members, some of whom may belong to a majority racial group. In other words, we do not know if this effect is explained by a broad ingroup-outgroup relationship or if these findings only characterize behaviors directed towards racial minority or low status group members from racial majority or high status group members. Moreover, the majority of our participants were from upper-middle to upper-class families, and there is some evidence to suggest that social class may also influence egalitarian behaviors (Piff et al., 2010). To this point, our findings mark the need for future work to continue to investigate the intersections of intergroup behavior and perceived group status (Rutland et al., 2010). Clearly, future research needs to examine participants from more diverse backgrounds in order to fully understand the role that adult-like, normative perspectives about race may play in egalitarian behaviors towards outgroups.

Relatedly, the present work also only examined egalitarian preferences for White and Black partners. We selected these groups because of the history of Black–White relations in the United States, which makes a Black phenotype a highly salient racial category for White children and one around which they readily form implicit biases (Baron and Banaji, 2006). However, it is unclear whether gender and racial constancy beliefs would affect sharing preferences in the same ways for other racial outgroups. Furthermore, it would be particularly important to examine three-way interactions among participant gender, racial constancy acquisition, and sharing partner race, since research should consider the intersectionality between race and gender more often to provide a more complete picture of the treatment of both gender and racial minorities. Unfortunately, we did not have sufficient power to examine this issue, and we felt it wise not to pursue these analyses given that interactions are notoriously underpowered in psychology research (Shaw, 2013).

Furthermore, we also know that children are sensitive to the relative status of both themselves and their partners (Sigelman and Waitzman, 1991; Raabe and Beelmann, 2011). For example, past work has shown that 5-year-olds are aware of wealth disparities in the United States (Weinger, 1998). Importantly, learned status perceptions of a group can significantly affect children’s social behaviors (Bigler et al., 2001; Fehr et al., 2008). Specifically, children may be likely to *give more* to those perceived as less fortunate (Li et al., 2014; Shaw et al., 2016); that is, children are inclined to redress inequities by sharing resources. Race, as a societally salient and significant social category (as opposed to a minimal group category) entails real-world implications (e.g., notions of status). Because racial group boundaries, at least in the United States, are deeply tied to status differences in the real world, racial constancy may consequently be associated with learning about group status differences (Huntsman, 1984; Nesdale and Flesser, 2001; Bigler et al., 2003; Pauker et al., 2010). For instance, kindergarteners and first graders more easily associate White individuals with being high status (Radke and Trager, 1950), 3–10 year olds are less likely to associate Black individuals with being high status (Olson et al., 2012), and children view Black individuals as lower in socio-economic status (Verkuyten and Masson, 1995). While the acquisition of racial constancy may be theoretically linked to children learning about group status differences, it remains to be assessed empirically.

## Conclusion

In sum, we examined whether both gender and gaining fixed, adult-like thinking about race predict egalitarian behaviors with racial ingroup versus outgroup members. Adding to the limited but growing literature regarding racial constancy attainment and its downstream consequences on racial perceptions (Semaj, 1980; Ruble et al., 2004; Pauker et al., 2010; Gaither et al., 2014) our findings are among the first to show that adopting a fixed outlook on race may alter egalitarian behaviors, at least for White children in certain contexts. While this does affect cross-race egalitarian perspectives in particular, our findings partially suggest a general increase in egalitarian behaviors toward one’s racial ingroup with the onset of adult-like thinking about race. Again, we caution the over-extrapolation of these findings given that they were only observed in one game of the three tested, but the one game with these results is the most directly related to intergroup behaviors related to inequality aversion – the original goal of this paper. Furthermore, we found evidence for gender effects in one Game, which replicates previous findings that girls display greater egalitarian behaviors than boys do. These findings point to potential social-developmental factors that may influence egalitarian behaviors at least for White children, and they highlight the need to test standardized developmental measurements such as egalitarian beliefs with more diverse stimuli and methods.

## Data Availability Statement

All datasets generated for this study are included in the article/supplementary material.

## Ethics Statement

The studies involving human participants were reviewed and approved by the Tufts University Institutional Review Board. Written informed consent to participate in this study was provided by the participants’ legal guardian/next of kin.

## Author Contributions

SG and SD designed and completed the initial study. SG completed all the data collection efforts. JP, SG, and SD analyzed the data and completed and wrote the publication. All authors approved the final version of this manuscript.

## Conflict of Interest

The authors declare that the research was conducted in the absence of any commercial or financial relationships that could be construed as a potential conflict of interest.
